# Pterostilbene-Induced Tumor Cytotoxicity: A Lysosomal Membrane Permeabilization-Dependent Mechanism

**DOI:** 10.1371/journal.pone.0044524

**Published:** 2012-09-05

**Authors:** Salvador Mena, María L. Rodríguez, Xavier Ponsoda, José M. Estrela, Marja Jäättela, Angel L. Ortega

**Affiliations:** 1 Green Molecular, Valencia, Spain; 2 Department of Physiology, University of Valencia, Burjassot, Spain; 3 Department of Cell Biology, University of Valencia, Burjassot, Spain; 4 Apoptosis Department and Centre for Genotoxic Stress Response, Institute for Cancer Biology, Danish Cancer Society, Copenhagen, Denmark; Vanderbilt University, United States of America

## Abstract

The phenolic phytoalexin resveratrol is well known for its health-promoting and anticancer properties. Its potential benefits are, however, limited due to its low bioavailability. Pterostilbene, a natural dimethoxylated analog of resveratrol, presents higher anticancer activity than resveratrol. The mechanisms by which this polyphenol acts against cancer cells are, however, unclear. Here, we show that pterostilbene effectively inhibits cancer cell growth and stimulates apoptosis and autophagosome accumulation in cancer cells of various origins. However, these mechanisms are not determinant in cell demise. Pterostilbene promotes cancer cell death via a mechanism involving lysosomal membrane permeabilization. Different grades of susceptibility were observed among the different cancer cells depending on their lysosomal heat shock protein 70 (HSP70) content, a known stabilizer of lysosomal membranes. A375 melanoma and A549 lung cancer cells with low levels of HSP70 showed high susceptibility to pterostilbene, whereas HT29 colon and MCF7 breast cancer cells with higher levels of HSP70 were more resistant. Inhibition of HSP70 expression increased susceptibility of HT29 colon and MCF7 breast cancer cells to pterostilbene. Our data indicate that lysosomal membrane permeabilization is the main cell death pathway triggered by pterostilbene.

## Introduction

Polyphenols form an extensive group of natural molecules with potential benefits for human health, e.g. cardiac protection, anticancer activities and diabetes control. The mechanisms underlying their anticancer activity have been extensively studied and include anti-oxidative, pro-apoptotic, pro-autophagic, DNA damaging, anti-angiogenic, and immunostimulatory effects. Resveratrol (Res), the most investigated polyphenol, shows a clear anticancer activity by both apoptotic and nonapoptotic mechanisms [1]. However, its low bioavailability (e.g. a 14.4 minutes half-life in the rabbit blood circulation after intravenous administration of 20 mg/kg) strongly limits its *in vivo* use [2]. Pterostilbene (3,5-dimethoxi-4′-hydroxystilbene; Pter) is a natural analogue of Res found in different plants such as grapes, blueberries and narra tree [3]. This natural phenolic phytoalexin, whose half-life in the blood stream is approximately five times higher than that of Res, displays potent fungicidal activity and similar or even more potent antitumor activitiy than Res [4].

The main problem in cancer treatment is to remove pools of cells capable of developing immunogenic, chemotherapeutic, and radiotherapeutic resistance. Apoptotic evasion represents one of the major obstacles since many classical antitumor agents trigger caspase-mediated apoptotic cell death [5]. Accordingly, new antitumoral strategies based on nonapoptotic mechanisms have been developed in the last years, and autophagy, lysosomal cell death and necrosis have emerged as alternative cell death programs in cancer cells [6]–[8].

Lysosomal volume and trafficking, as well as the expression of lysosomal hydrolases, are notoriously altered in transformed cells [9]. In fact, effective invasive growth and neoangiogenesis depend on these alterations [10]. Moreover, development of multidrug resistance has been also linked to the alterations in the endosome-lysosomal compartment [11]. In this sense, agents targeting lysosomes may provide means to revert multidrug resistance. Furthermore, lysosomal membrane permeabilization has recently emerged as an effective inducer of caspase-dependent and -independent cell death [12], and several inducers of lysosomal membrane permeabilization are presently under development as anti-cancer drugs. For example, siramesine (a piperidine analogue originally developed as an antidepressant) and BAMLET (a complex of bovine lactalbumin and oleic acid), can trigger lysosomal membrane permeabilization and non-apoptotic cell death even in apoptosis resistant cancer cells [7], [13].

Apoptosis and autophagy have been postulated as main cell death mechanisms induced by Res i.e., [1], [14], [15]. Nevertheless, the anticancer mechanisms induced by Pter remain unclear. In agreement with other studies we show here that Pter induces growth arrest and cancer cell death [16]. Although Pter can activate apoptosis, we show that this pathway is not essential for its cytotoxic effect. Our results show that Pter induces destabilization of the lysosomal membranes and release of lysosomal hydrolases into the cytoplasm, thus leading to the activation of a caspase-independent lysosomal cell death program.

## Materials and Methods

### Cell Culture and Treatments

The cell lines were purchased from the American Type Culture Collection (ATCC). Human melanoma A375, human lung carcinoma A549, and human colon adenocarcinoma HT29 were cultured in Dulbecco’s modified Eagle’s medium (DMEM, Invitrogen), and human breast adenocarcinoma MCF-7 in RPMI-1640 medium (Invitrogen). The mediums were supplemented with 10% heat-inactivated fetal bovine serum (FBS, Invitrogen), 100 units/ml penicillin (Invitrogen), and 100 µl/ml streptomycin (Invitrogen). Cells were grown under standard cell culture conditions (37°C, 5% CO_2_/95% air atmosphere) at a density of 20,000 cells/cm^2^.

Cancer cells were plated and allowed to attach for 24 h. Then cells were treated with Res (Sigma-Aldrich) or Pter (Green Molecular) for 24, 48, and/or 72 h.

### Cell Growth Analysis and IC_50_ Determination

Cell growth was analyzed using the Countess Automated Cell Counter (Invitrogen). To evaluate IC_50s_ in the presence of Pter or Res we used the Sulphorhodamine B Toxicology Assay Kit following manufacturer’s instructions (Sigma-Aldrich) and U.S. NCI recommendations. Briefly, cells were seeded in 96-well plates (5,000 cells/well) and treated with Pter or Res (Sigma-Aldrich) (0–200 µM). Forty-eight hours after polyphenols addition cells were fixed with 10% trichloroacetic acid. Cell viability was determined by cellular staining with sulforhodamine B (0.4%) in acetic acid (1%). Viability data were represented in semilog dose-response curves. The IC_50_ values were calculated with Prism GraphPad software, where the IC_50_ value represents the concentration of the test item that induces a response halfway between the baseline and a maximum response.

### Flow Cytometric Analyses

Cells were treated with polyphenols for 24 h, washed in phosphate buffered saline (PBS), trypsinized, collected with cold PBS, pelleted (1,000 g for 2 minutes), fixed with 70% chilled ethanol, and kept at 4°C overnight. Then these cells were pelleted and washed once in PBS before DNA staining with propidium iodide (Sigma-Aldrich), in the presence of RNase for 15 minutes.

Analysis of apoptotic and necrotic cell death was carried out according to manufacturer instructions using Annexin V Alexa Fluor 488 (Invitrogen) and propidium iodide. Briefly, cancer cells were seeded and, 24 h later, were treated with different concentrations of Pter or Res. After 48 h in the presence of polyphenols cells were harvested and washed once in PBS, then incubated with a solution containing Annexin V Alexa Fluor 488 in a buffer containing propidium iodide.

Lysosomal membrane integrity was evaluated using acridin orange. In these experiments after Pter treatment (see above) cells were stained with 2 µg/ml acridine orange (Invitrogen) which was present in the culture medium for 15 minutes.

The DNA cellular content, cell death analysis and lysosomal membrane integrity was analyzed with a BD FACSCanto II flow cytometer (10,000 cell events were collected per sample).

### 5-Bromo-2′-Deoxyuridine Incorporation Assay

Proliferation assays were done using the Roche Cell Proliferation ELISA BrdU kit according to manufacturer’s instructions. Cells were plated in 96-well microplates and, 24 h later, treated with Pter for 4 h. Then, cells were labeled by the addition of BrdU (10 µM/well) for another 2 h. After that, cells were fixed and DNA was denatured with FixDenat solution (200 µl for 30 minutes) at room temperature. Finally, antiBrdU-POD solution was added for 90 minutes. Using a TMB-like substrate, immune complexes were detected by measuring the absorbance at 370 nm and 492 nm using a Multiskan® Spectrum (Thermo scientific).

### Caspase Activity Assay

Detection of caspase-3/7 activity was performed with the Apo-One Homogeneous Caspase-3/7 Assay Kit (Promega) according to manufacturer instructions. The assay was configured for 96 well plates (5,000 cells/well). Capase-3/7 activities were evaluated 24 and 48 h after polyphenols addition. This assay provides a profluorescent substrate (Z-DEVD-R110) which is cleaved by caspase-3/7 activity to generate the fluorescent rhodamine 110. Fluorescence was measured by a Fluoroskan Ascent FL (Thermo Labsystems) (excitation at 485 nm; emission at 538 nm).

### Protein Extraction and Western Blot Analysis

After experimental treatments cells were rinsed twice with cold PBS. Then, proteins were extracted from cultured cells by scraping in ice-cold lysis buffer (CelLytic™ MT Mammalian Tissue Lysis/Extraction Reagent, Sigma-Aldrich) containing a protease and phosphatase inhibitors cocktail (Sigma-Aldrich). Equal amounts of extracted proteins (30 µg/condition) were resolved using a criterion XT bis-tris 4–12% gel (Bio-rad) and transferred to an Immun-Blot™ PVDF membrane (Invitrogen). Blots were incubated overnight at 4°C, separately, with the primary antibodies: Rabbit polyclonal LC3A/B antibody (1∶500) (Abcam); rabbit monoclonal GAPDH antibody (1∶1,000) (Cell signaling); rabbit monoclonal p62 antibody (1∶1,000) (Sigma-Aldrich); rabbit monoclonal HSP70 antibody (1∶200) (Enzo Life Science). Blots were incubated for 1 h at room temperature with the appropriate peroxidase-conjugated secondary antibody: Anti-rabbit IgG HRP-linked antibody (1∶1,000) (Cell signaling). Blots were visualized using a chemiluminescence detection kit ECL western blotting substrate (Pierce, Thermo Scientific) and the signals were captured using a ChemiDoc™ XRS+Imaging System (Bio-Rad). The density of the bands was measured using Image Lab Software™ version 2.0.1 (Bio-Rad).

### Confocal and Fluorescence Microscopy

A375-, A549-, MCF7-, and HT29-GFP-LC3 cells were seeded out on coverslips in 24-well plates and allowed to attach for 24 h. Then cells were treated with Pter (50 µM) for 24 h, or with Concanamycin A (2 nM) (Sigma-Aldrich) or Rapamycin (100 nM) (Sigma-Aldrich) for 1 h. Both control and treated cells were fixed with paraformaldehyde (PFA) 4% for 10 minutes at 4°C. Cells were washed twice with DPBS (Invitrogen), mounted on microscope slides with ProLong Gold antifade reagent (Invitrogen), and, then, were visualized using a confocal laser microscope (Leica TCS Sp2).

Lysosomal visualization was carried out after Pter treatment using the fluorophore Lysotracker Red (Invitrogen). Briefly, cells were seeded in 6-well cell culture plates. After 24 h of treatment with Pter cells were stained according to manufacturer recommendations (75 nM, 30 minutes) and imaged with a Nikon Diaphot inverted microscope.

### Lactate Dehydrogenase Assay

Cytotoxicity induction was determined by measuring lactate dehydrogenase (LDH) activity released to the extracellular medium. The LDH release assay was performed using a citotoxicity detection kit^plus^ (LDH) (Roche Diagnostics) according to the manufacturer’s instructions. This kit determines spectrophotometrically the amount of reduced formazan at 490 nm. This measurement was done with a Multiskan Spectrum (Thermo scientific). The LDH content of each sample was calculated according to the following formula: Cytotoxicity (%) = [(experimental value − low control)*/*(high control − low control)] × 100.

### Cathepsin Activity Assays

Cancer cell lines were seeded in six well-plates (0.2×10^6^ cells/well) and, 24 h later, were treated with Pter (0–100 µM). After removal of the medium, extraction buffer containing different concentrations of digitonin (Sigma-Aldrich) was used to separate cytosolic (20 µg/ml) and total (200 µg/ml) cathepsins. When necessary, the concentration of digitonin was optimized for different cell types. Cells were incubated with extraction buffer for 15 minutes at 4°C on a rocking platform. Cysteine and aspartate cathepsin activities were measured using the fluorescent substrates z-fr-AFC (AFC = 7-Amino-4-trifluoromethylcoumarin) (excitation at 405 nm; emission at 510 nm) (Enzo Life Sciences) and Mca-GKPILFFRLK(Dnp)-DR-NH_2_ [Mca =  (7-methoxycoumarin-4-yl)acetyl; Dnp = dinitrophenyl] (excitation at 320 nm; emission at 420 nm) (Enzo Life Sciences), respectively. Pepstatin A (5 µg/ml) (Sigma-Aldrich) and Leupeptin (50 µg/ml) (Sigma-Aldrich) were used to inhibit the activity of aspartyl peptidases and serine-cysteine proteases, respectively.

### Electron Microscopy

Cell monolayers were fixed with 2% glutardaldehyde in 0.1 M phosphate buffer, pH 7.4, for 30 minutes, and with 2% osmium tetroxide in water for 1 h. Then, samples were dehydrated with ethanol and counterstained with uranyl acetate, and finally embedded in TAAB resin (T002, Taab Laboratories). Cells were sectioned at 2 µm and stained with toluidine blue. Selected semithin sections were re-embedded and trimmed for ultrathin sectioning. Ultrathin sections were stained with lead citrate and examined in a Jeol JEM-1010 electron microscope.

### siRNA Transfection

Cells were transfected with siRNA targeting HSPA1A and HSPA1B (ON-TARGETplus SMARTpool, Dharmacon Research, USA) at a final concentration of 25 nM per well. As positive control we used a siRNA against glyceraldehyde phosphate dehydrogenase (ON TARGETplus GAPDH Control Pool, Dharmacon Research, USA). To account for the nonsequence-specific effects, siControl Non-Targeting Pool from Dharmacon was used.

MCF7 and HT29 cell lines were seeded on 12-well plates (75,000 cells/well) one day prior to transfection. DharmaFECT 1 siRNA Transfection Reagent (3 µl) was added to 98 µl of serum free medium and incubated at room temperature for 5 minutes. Hsp70, negative or positive control siRNA (100 µl) were added and incubated for another 20 minutes. The transfection reagent/siRNA complex was mixed with 1 ml of DMEM with 10% FBS and added to the cells. After siRNA addition, the cells were incubated at standard culture conditions (37°C, 5% CO_2_).

### Statistical Analyses

Values are expressed as the mean ± SD. All experiments were repeated at least three independent times. Data were analyzed using the Graph Pad Prism version 5.00. The significance of the difference between the control and each experimental test condition was analyzed by Student’s t test or one way ANOVA followed by a Tukey-test. A value of P<0.05 was considered significant.

## Results

### Pterostilbene Shows Higher Anticancer Potential than Resveratrol

**Figure 1:**
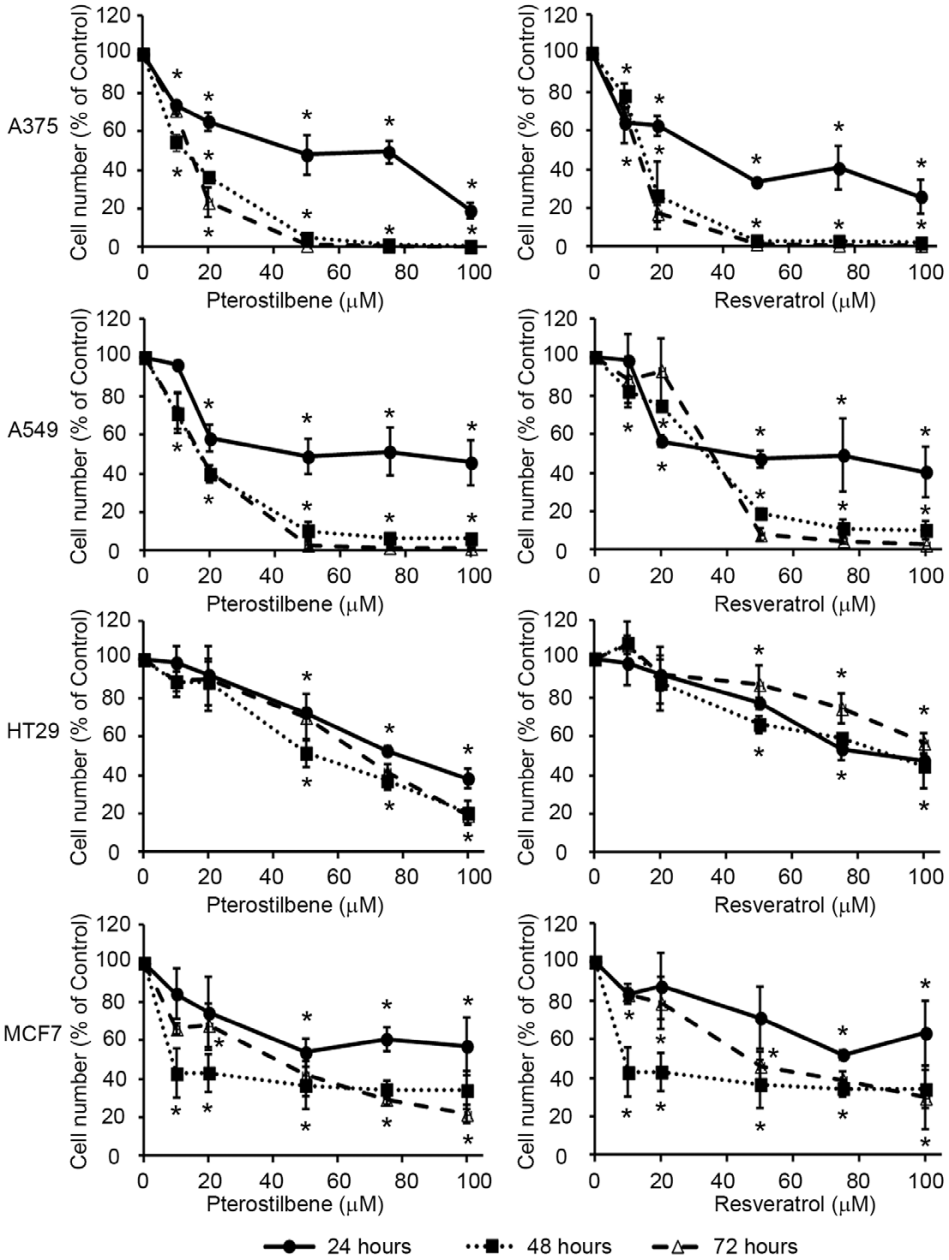
Effect of pterostilbene and resveratrol on tumor cell growth *in vitro*. A375, A549, HT29 and MCF7 cells were incubated for 24, 48 or 72 h with indicated concentrations of Pter or Res. Actual cell number was analyzed using the Countess Automated Cell Counter. Results were expressed as relative proliferation index ± SD (n = 4) where control is 100% (*P<0.05).

Structural modifications of the Res molecule can increase its *in vivo* half-life without causing toxic side-effects in normal tissues [17]. Treatment of human A375 (melanoma), MCF7 (breast adenocarcinoma), A549 (lung cancer) and HT29 (colon cancer) cells with Pter reduced tumor cell number *in vitro* in a concentration- and time-dependent manner (Figure 1). IC_50s_ determined by Sulphorhodamine B showed that the growth inhibitory effect of Pter was cell type-dependent, being much lower for HT29 (Pter IC_50_ = 60,3 µmol/L; Res IC_50_ = 71,9 µmol/L) and MCF7 (Pter IC_50_ = 44,0 µmol/L; Res IC_50_ = 56,6 µmol/L) cells than for A375 (Pter IC_50_ = 14,7 µmol/L; Res IC_50_ = 25,5 µmol/L) and A549 (Pter IC_50_ = 28,6 µmol/L; Res IC_50_ = 36,2 µmol/L) cells. Notably, the IC_50_ values were lower for Pter than for Res in all cases.

### Pterostilbene Induces Cell Cycle Arrest and Blocks DNA Synthesis

**Figure 2:**
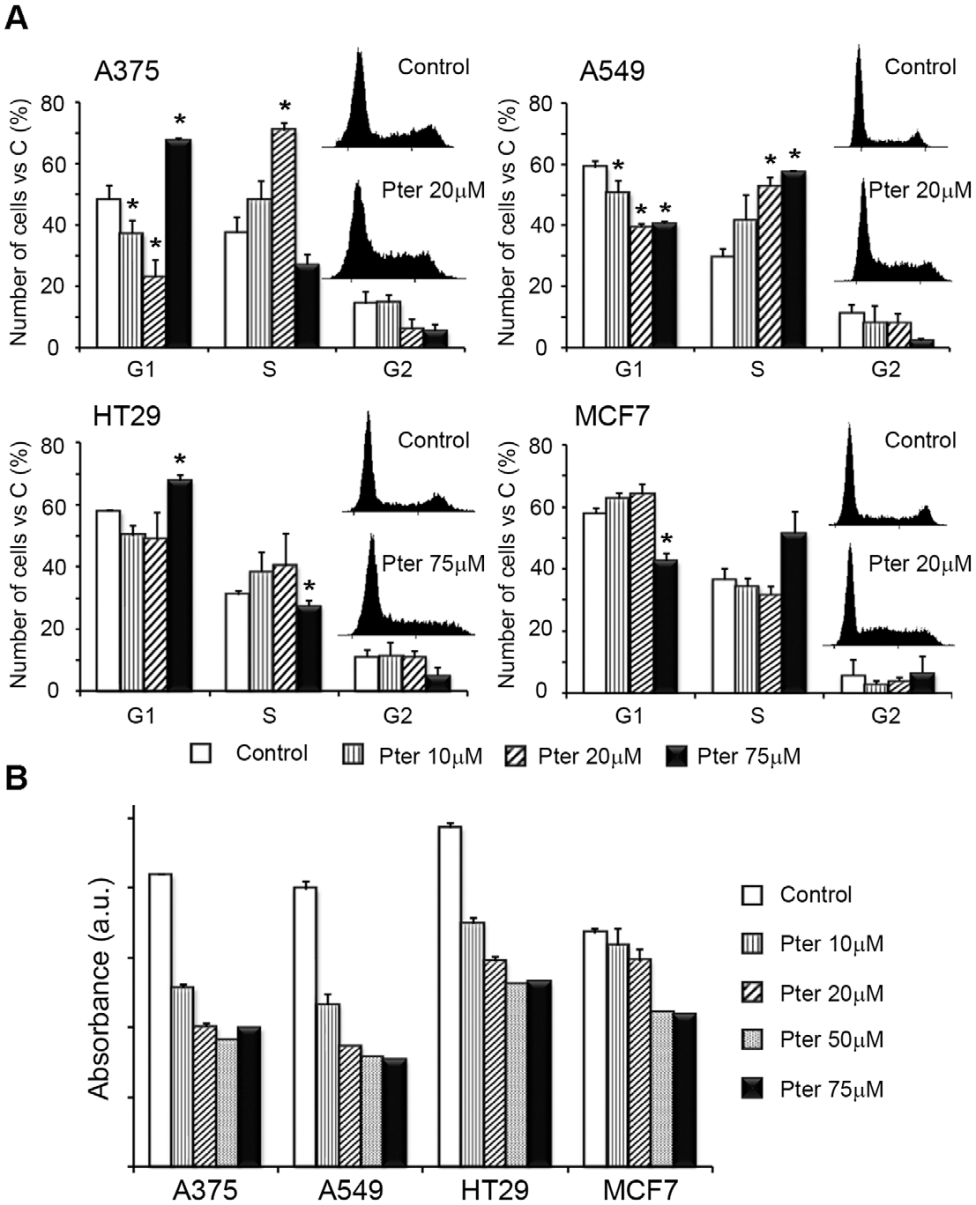
Effect of pterostilbene on cell cycle distribution and DNA synthesis. A) Tumor cells were seeded in six well-plates and treated 24 h later with indicated concentrations of Pter. After 24 h, cells were fixed and stained with propidium iodide as explained under material and methods. The DNA content of the cells was analyzed by flow cytometry (10,000 cell events were collected per sample). Representative cell cycle histograms for Control and Pter-treated cells are shown. Results are expressed as % of total cells ± SD (n = 3) (*p<0.05 Vs. Control). B) DNA synthesis was evaluated by BrdU incorporation after 4 h of incubation with Pter followed by 2 h with Pter and 10 µM BrdU.

Microscopic analysis (data not shown) and data presented above clearly showed that Pter induced cell death in all cancer cell lines studied. In order to test whether the cell death was preceded or accompanied by a growth arrest, we analyzed the effect of Pter on cell cycle distribution at the 24 h time point. A375 and A549 cells showed an arrest in the S phase of the cell cycle after 24 h of treatment with 20 µM Pter (Figure 2A). A similar S phase arrest was observed in HT29 cells upon treatment with 75 µM Pter, and also MCF7 cells treated with 10–20 µM Pter showed a tendency to arrest in the S phase (Figure 2A). Notably, higher concentration (75 µM) of Pter significantly reduced the portion of A375 cells in the S phase suggesting that the cells arrested in the S phase before succumbing (Figure 2A).

To evaluate the effect of Pter on DNA synthesis we measured the ability of cells to incorporate BrdU after 4 h incubation with increasing concentrations of Pter. Akin to earlier reports on Res and other polyphenols [14], [15], [18], Pter reduced the BrdU incorporation in a concentration-dependent fashion in all cell lines.

### Cell Death Induction by Pterostilbene is Time- and Concentration-Dependent

The molecular mechanisms underlying the anticancer effects of natural polyphenols are poorly defined. Res has been shown to be a potent inducer of both apoptosis [1], [14] and autophagy [1], [15], as well as a carcinogenesis inhibitor [19], [20]. However, anticancer mechanisms of its dimethylated analog, Pter, have not been extensively analyzed. For this purpose we investigated the ability of Pter ability to induce apoptosis, autophagy and necrosis.

**Figure 3:**
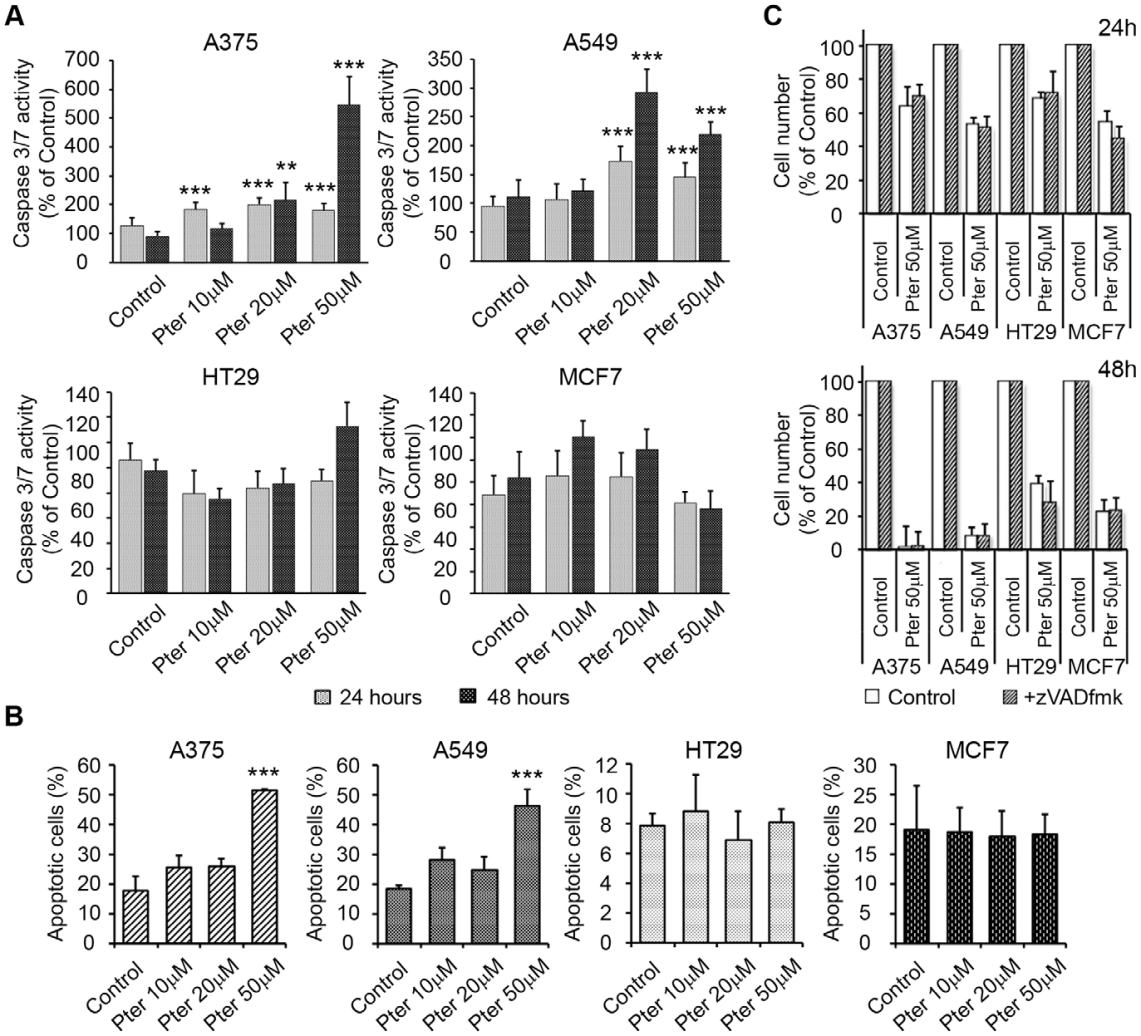
Pter induces caspase-independent cell death. A) Caspase-3/7 (DEVDase) activities were evaluated 24 and 48 h after addition of Pter. Results are expressed as a relative index of fluorescence ± SD (n = 3) when compared with control (100%). The results were statistically analyzed by ANOVA and contrasted by the Tukey test (*p<0.001; **p<0.01 Vs. Control). B) Induction of apoptosis was assayed by staining with Annexin V. The total percentage of Annexin V-positive cells was determined by flow cytometry 48 h after treatment with DMSO or with increasing concentrations of Pter (*p<0.001 Vs. Control). C) Effect of Pter on tumor cell number in the presence of 20 µM pancaspase inhibitor zVAD fmk, which was added 1 h prior to the addition of 50 µM Pter or vehicle (1.5 µl/ml DMSO). Cell number was analyzed 24 and 48 h later. Results are expressed as relative proliferation index ± SD (n = 3) where control is 100%.

First, we measured the activity of apoptotic effector caspases 3 and 7 (DEVDase activity) in cancer cells treated with Pter. Pter induced a significant caspase 3/7 activation in A375 and A549 cells, but not in HT29 and MCF7 cells (Figure 3A). Accordingly, Pter induced the translocation of annexin V to the cell surface (marker of apoptosis) only in A375 and A549 cells (Figure 3B). To evaluate the contribution of apoptosis on cell death induction we used a cell-permeable pan-caspase inhibitor z-VADfmk. Notably, z-VAD-fmk failed to inhibit the Pter-induced reduction in cell number (Figure 3C). Positive controls of apoptosis and zVADfmk inhibition capability are shown in Supporting Information (Figure S1). These results suggest that caspase-independent mechanisms may play a role in Pter-induced cytotoxicity.

**Figure 4:**
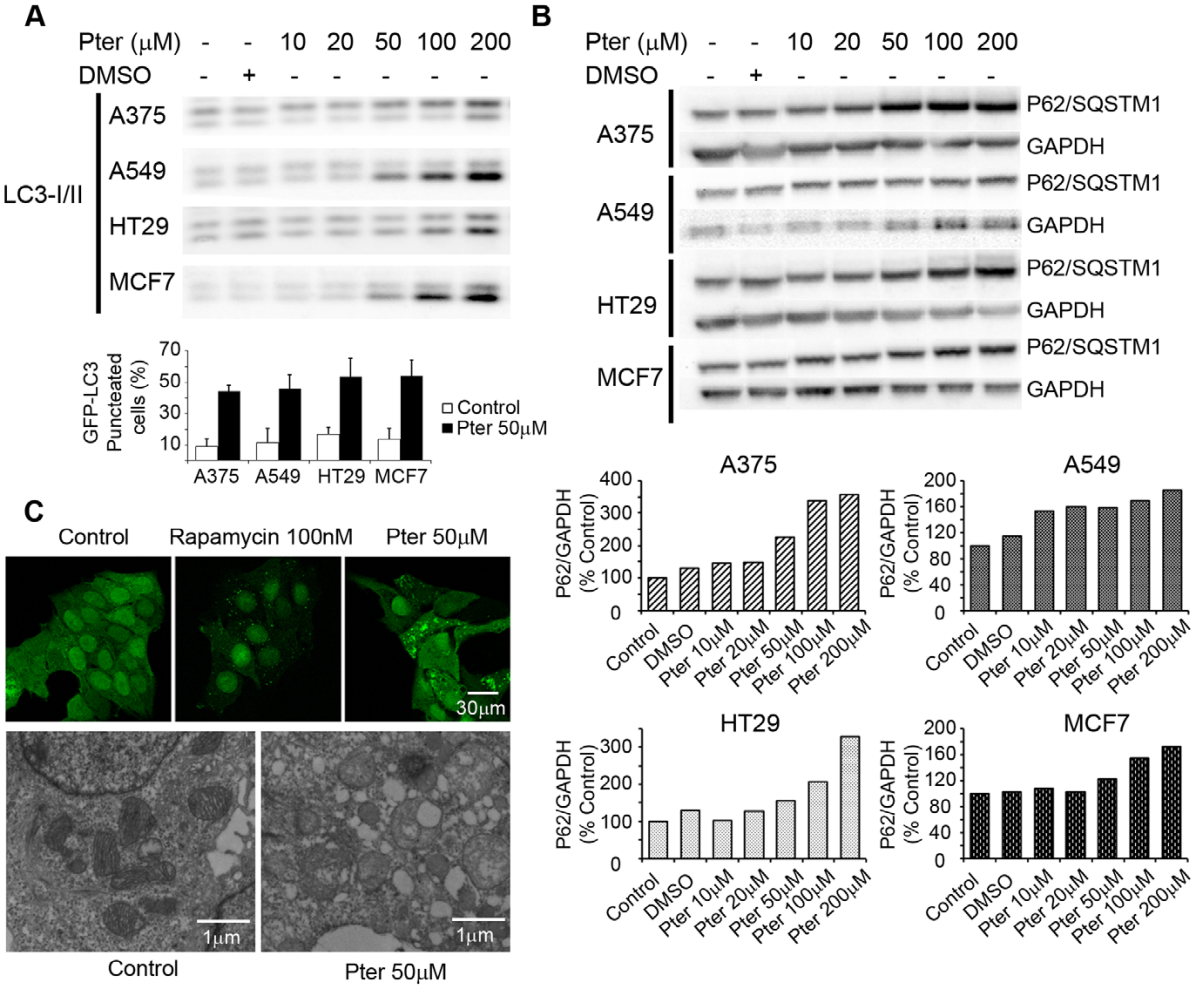
Effects of pterostilbene on autophagic flux. Proteins were detected by western blot with LC3A/B (A) or SQSTM1/p62 antibodies (B). Graphic shows the percentage of induction of eGFP-LC3 punctuation/aggregation in control and Pter (50 µM) conditions after 24 h of treatment (A). Densitometric analysis of SQSTM1/p62 correlated with GAPDH levels. The ratio in untreated control cells was set to 100% (B). MCF7-eGFP-LC3 cells were visualized by confocal and transmission electronic microscopy after treatment with 50 µM Pter for 24 h or 100 nM Rapamycin for 1 h (C).

To investigate the involvement of autophagy in Pter-induced cytotoxicity, we analyzed the levels of LC3II and P62/SQSTM1 that should increase and decrease, respectively, upon autophagy induction [21], [22]. Notably, 24 h treatment with Pter induced a concentration-dependent accumulation of both LC3II (Figure 4A) and P62 (Figure 4B) proteins in all four cell lines as analyzed by immunoblotting. Furthermore, confocal and fluorescence microscopy of A375, A549, MCF7, and HT29 cells expressing a GFP-tagged LC3 revealed a clear increase in the number of large LC3-positive vesicles upon 24 h treatment with Pter (Figures 4A bottom, and 4C). Visualization of numerous large (diameter up to 2 µm) vesicles surrounded by a double membrane by transmission electron microscopy confirmed that these vesicles are enlarged autophagosomes (Figure 4C). These results are suggestive of autophagosome accumulation due to inhibited autophagic flux [22].

**Figure 5:**
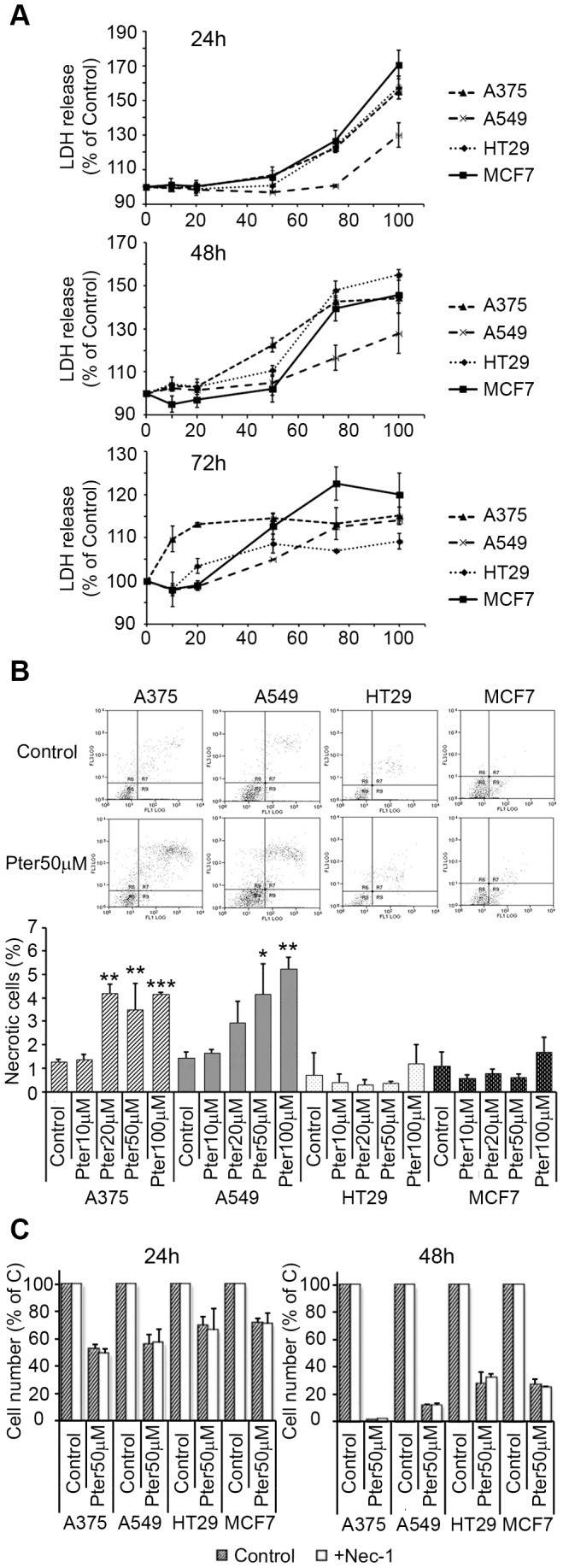
Effect of pterostilbene on necrosis induction. A) Release of LDH activity to the extracellular medium. Cells were exposed to Pter for 24, 48 and 72 h. Results are expressed as relative LDH activities ± SD (n = 3) where control was set to 100%. B) Propidium iodide fluorescence was plotted against annexin-V fluorescence 24 h after treatment. Results are expressed as total percentage of necrotic cells ± SD (n = 3). C) Effect of Pter on tumor cell number in the presence of the necroptosis inhibitor 5-(Indol-3-ylmethyl)-(2-thio-3-methyl)hydantoin (Nec-1, Calbiochem). Nec-1 25 µM was added 1 h prior to the addition of 50 µM Pter. Cell number was analyzed 24 and 48 h later. Results are expressed as relative proliferation index ± SD (n = 3) where control is 100%. The results were statistically analyzed by ANOVA and contrasted by the Tukey test (***p<0.001; **p<0.01; *p<0.05 Vs. Control).

To determine the ability of Pter to induce necrotic cell death, we investigated the integrity of the plasma membrane by measuring the release of cytosolic lactate dehydrogenase (LDH) to the culture supernatant [23]. The LDH release was stimulated in all cell lines after 24 h treatment with Pter at concentrations≥50 µM (Figure 5A). Akin, flow cytometry analyses with Annexin V-FITC and propidium iodide showed just a moderate necrosis increase in A375 and A549 (Figure 5B). Although, LDH release was increased after 48 and 72 h of Pter treatment (Figure 5A), inhibition of necroptosis, a serine–threonine kinase receptor-interacting protein 1 (RIP1) dependent form of necrosis [24], did not affect Pter-induced cell death (Figure 5 C). Thus indicating that other type of cell death induction different of apoptosis, autophagy, and necrosis may be activated by Pter. Positive controls showing the capability of necrostatin-1 to inhibit necroptotic cell death are shown in Supporting Information (Figure S2).

### Pterostilbene Induces Lysosomal Membrane Alterations

**Figure 6:**
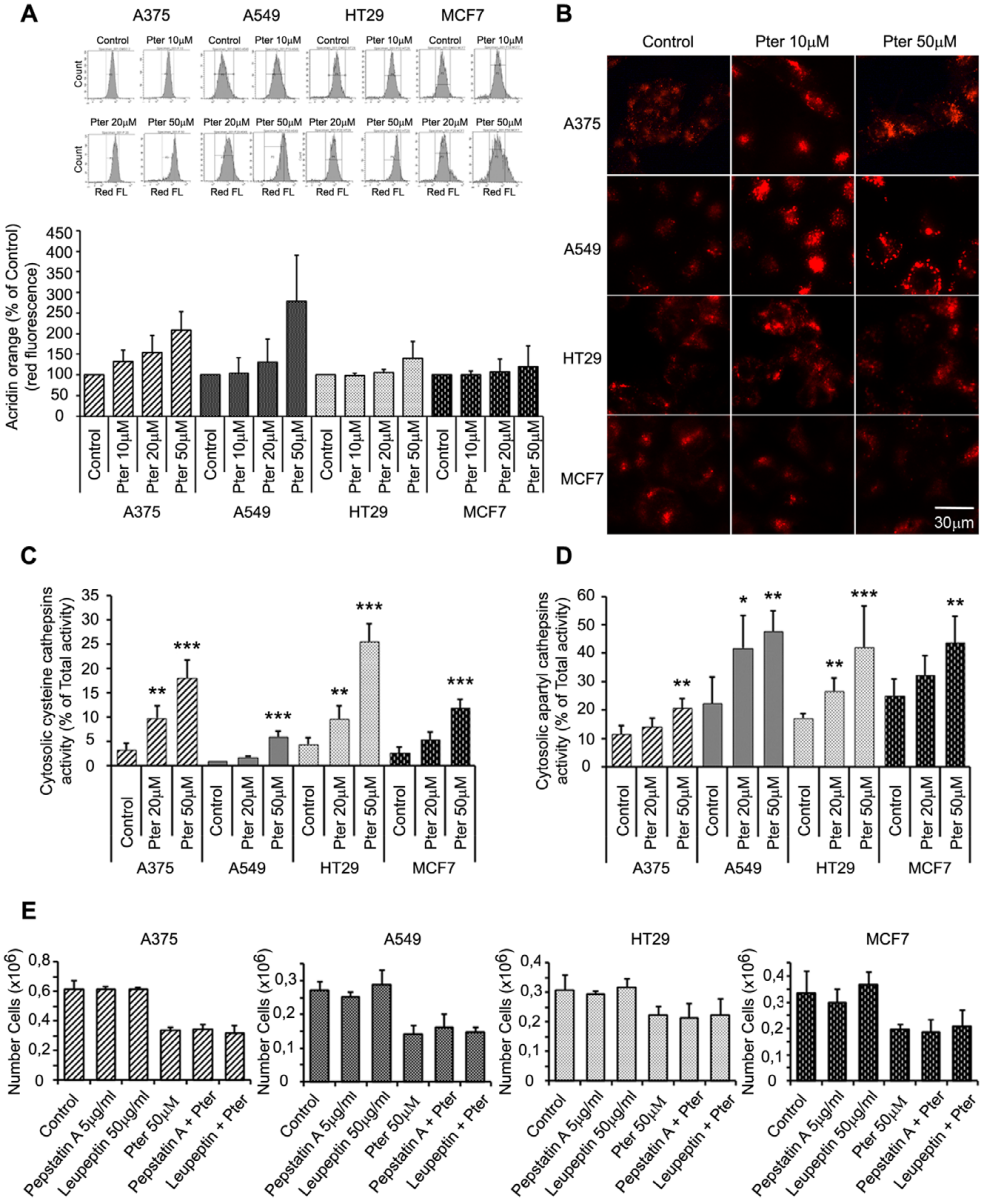
Pterostilbene induces lysosomal membrane permeabilization. A) Flow cytometry analysis of acridine orange staining after 24 h incubation with indicated concentrations of Pter. Increases in red fluorescence (x-axis), observed in treated cells, indicated changes at the lysosomal membrane. B) Microscopic images of Lysotracker red-stained cells untreated or treated with indicated concentrations of Pter for 24 h. C) Cytosolic cysteine cathepsin (zFRase) activity expressed as percentage of total activity (n = 7) was measured 24 h after Pter addition. D) Cytosolic aspartyl cathepsin activity expressed as percentage of total activity (n = 7) after 24 h of Pter treatment. The results were statistically analyzed by ANOVA and the Tukey test (*p<0.05; **p<0.01; ***p<0.001 vs. control). E) Cathepsins B and D inhibitors, Leupeptin and Pepstatin A, were added 1 h prior to the addition of 50 µM Pter. Cell number was analyzed 24 h later.

Lysosomal leakage has been described as an inducer of cell death [7], [25], and lysosomal destabilization has been shown to be a common consequence of microtubule-targeting drugs [26]. Thus, to further elucidate the mechanisms underlying Pter-induced cancer cell death, we studied its possible effects on lysosomes. For this purpose we evaluated lysosomal membrane alterations by different methods. First, lysosomal volume was assayed using acridine orange staining and flow cytometry 24 h after exposure to Pter. Acridine orange is a metachromatic fluorescent cationic dye that emits red light when in high concentration inside the lysosomes, and green light in the cytosol and nucleus where it mainly stains DNA and RNA and is less concentrated than in acidic lysosomes. We evaluated the red fluorescence as an indicator of lysosomal volume [27]. Significant increases in red fluorescence were observed in all tumor cell lines upon treatment with Pter 50 µM (Figure 6A). As shown by fluorescence microscopy of cells stained with lysotracker, Pter induced an increase in the number and/or size of lysosomes (Figure 6B) We observed that these changes were higher in A375 and A549 than in HT29 and MCF7 cells (Figure 6A, B).

Next, we analyzed the release of lysosomal cysteine (B, L and H) and aspartate (D and E) cathepsins to the cytosol as a marker of lysosomal membrane permeabilization. Remarkably, Pter caused a concentration-dependent increase in the cytosolic cathepsin activities in all four cell lines without affecting the total cellular activities after 24 h of treatment (Figure 6C, D). To determine the contribution of cysteine (B, L, and H) and aspartate (D, and E) cathepsins to cell death, we inhibited the activity of aspartyl peptidases with pepstatin A, and the serine cysteine proteases with leupeptin. As shown in Figure 6E Pter induced the same effect in the presence or absence of these enzyme inhibitors.

### Tumor Resistance to Pterostilbene Correlates with HSP70 Cellular Levels

**Figure 7:**
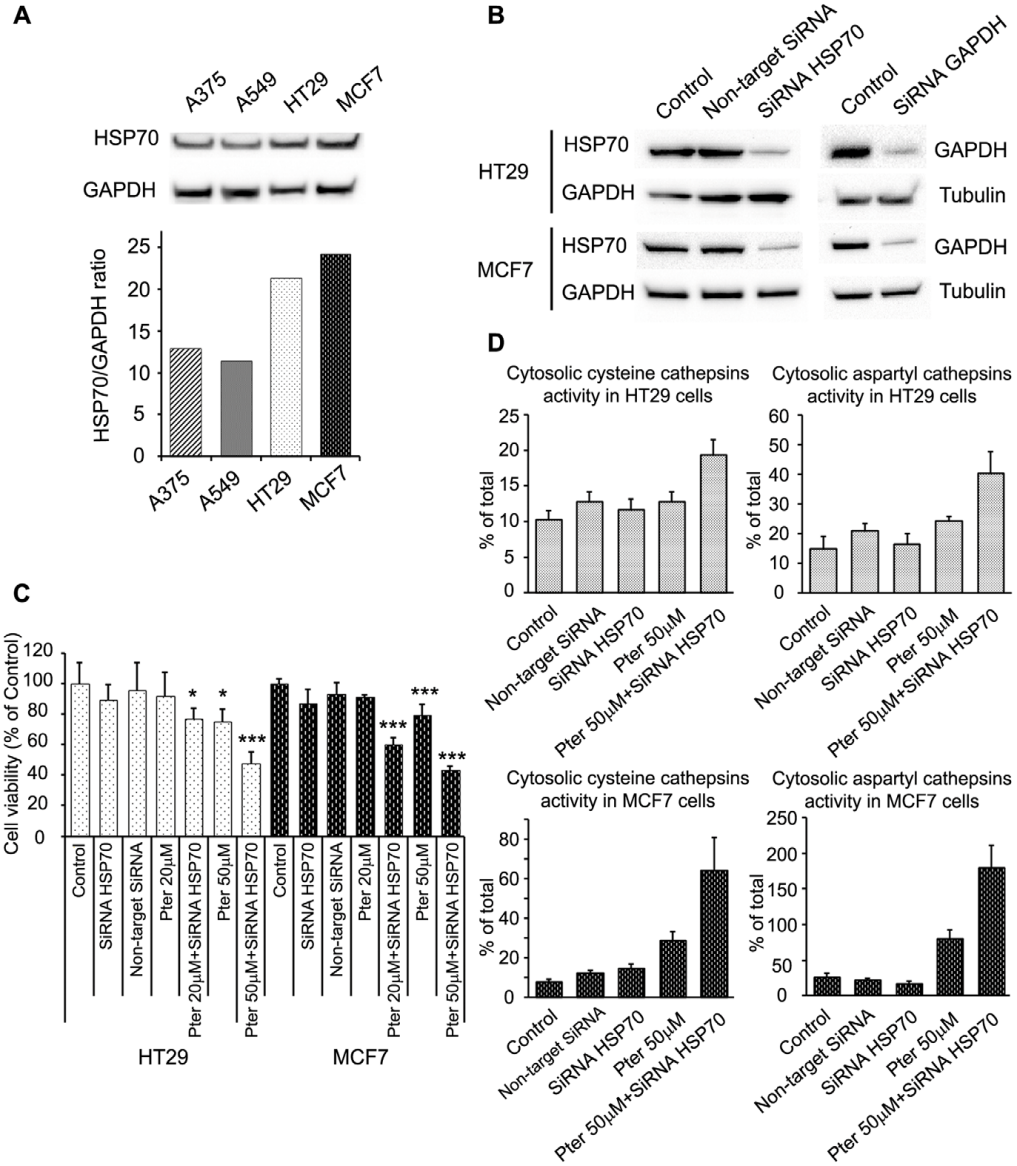
HSP70 depletion sensitizes cells to Pter. A) Representative HSP70 and GAPDH (loading control) immunoblots of proteins extracted from indicated cells. Densitometric analysis of the immunoblots (n = 3) are shown below. B) Inhibition of HSP70 expression by siRNA. Cells were transfected with HSP70 siRNA or GAPDH SiRNA (positive control). Protein levels were measured by western blotting. C) HT29 and MCF7 cells were seeded and transfected with HSP70 siRNA 24 h later. Pter was added at 24 h post-transfection. Cell viability was performed by sulforhodamine B colorimetric assay 48 h later. The number of replicates per experiment was at least six (n = 3). The results were statistically analyzed by ANOVA and the Tukey test (*p<0.05; ***p<0.001 vs. control). D) Cysteine and aspartyl cathepsins were determined in HT29 and MCF7 cells 72 h post- HSP70 siRNA transfection and 24 h after Pter treatment (n = 6). The results were statistically analyzed by ANOVA and the Tukey test (***p<0.001 vs. Pter 50µM).

Heat shock protein 70 (HSP70) is an evolutionary conserved protein that promotes the survival of stressed cells, where stabilization of lysosomal membranes is one of the protective mechanisms [28]. Different studies have shown a relationship between Res and HSP70 expression, although taking into account the protector role of the protein, HSP70 levels could regulate the distinct grade of susceptibility shown by the tumor cell lines. To characterize the higher resistance of HT29 and MCF7 to Pter treatment (Figure 1, Figure 2, Figure 3A, B and Figure 4C) we analyzed the possible involvement of HSP70. As shown in Figure 7A, Hsp70 levels were found to be higher in HT29 and MCF7 cells than in A375 and A549. Moreover, siRNA-mediated reduction of HSP70 in HT29 and MCF7 (Figure 7B) cells resulted in a marked cell death induction (Figure 7C) and a higher lysosomal membrane susceptibility to Pter (Figure 7D). Therefore, HSP70 levels, indeed, correlate with the sensitivity of the tumor cells to Pter-induced cytotoxicity.

## Discussion

Cancer is a genetic and epigenetic disease which is associated with unlimited replicative potential, reduced dependence on growth-promoting signals, resistance to growth-inhibitory signaling, evasion of cell death activation, sustained angiogenesis, as well as the ability for tissue invasion and metastatic spread [5]. Although new therapies may extend patients’ lives for days, months, or even years, the quality of life added is in most cases questionable. Thus, the development of therapies that could improve efficacy without compromising a reasonable quality of life, are urgently required. It is in this particular sense that natural polyphenols may offer some hope [29].

Anticancer mechanisms elicited by natural polyphenols have been extensively studied in the last years. However, reported effects claimed for polyphenols must be carefully evaluated since large differences, even controversial, are found depending on experimentals [29]. Res, one of the most studied polyphenols, shows the ability to induce apoptosis and autophagy, and has antiproliferative effects [1], [14], [15], [30], [31]. However, its antioxidant properties may potentially reduce oxidative stress thereby inhibiting apoptosis and enhancing cell survival [32]. Although potent anticancer effects have been shown in cultured cells, potential inhibition of cancer growth by Res *in vivo* is strongly limited due to its low bioavailability [2]. Therefore, it is important to take into consideration other chemical structures, which may preserve anticancer properties while having higher bioavailability.

It is in this context that Pter, a natural dimethylated analog of Res with a longer half-life [29], represent an attractive option. Pter showed higher anticancer effects than Res in all the tumor cell lines assayed (Figure 1). Nevertheless, A375 melanoma and A549 lung cancer cells were more susceptible to the anticancer action than HT29 colon cancer and MCF7 breast cancer cells. Thus suggesting that, the reduction in tumor cell number induced by Pter depends on differences in the cell lines assayed.

To assay if pterostilbene shows a preferential effect on cancer cell lines versus their normal counterparts we used normal human primary epidermal melanocytes from neonatal foreskin and epithelial lung cells (BEAS2B). The growth inhibitory effect *in vitro* was determined by Sulphorhodamine B and showed that Pter’s IC_50_ for melanocytes (Pter IC_50_ = 52,1 µmol/L) was similar to that found for breast (MCF7) and colon (HT29) cancer cell lines (see Results section). Whereas epithelial lung cells showed a much lower IC_50_ value (Pter’s IC_50_ = 1,8 µmol/L), but again similar to those found for A375 or A549 cells. Nevertheless, this is no surprise. Different polyphenols, such as e.g. resveratrol, curcumin, green tea polyphenols, or pterostilbene, have anticancer activity *in vivo* but without showing any relevant toxicity for the host at therapeutic effective doses (e.g. [4], [33], [34]). Naturally this apparent biological paradox is intriguing. However it seems obvious that under *in vivo* conditions other mechanisms, besides polyphenols (but related to), play a relevant role. In this sense the tumor microenvironment is of particular interest. For instance, in a previous report, we showed that intravenous administration to mice of pterostilbene and quercetin, two structurally related and naturally occurring small polyphenols, inhibits *in vivo* metastatic growth of highly malignant B16 melanoma F10 (B16–F10) cells [35].

Pterostilbene and quercetin inhibit bcl-2 expression in metastatic cells, which sensitizes them to vascular endothelium-induced cytotoxicity [35] (a physiological defense mechanism against metastatic cell invasion [36]). In addition polyphenols increased endothelial NO synthase expression in the vascular endothelium, thus favoring endothelial NO-induced tumor cell cytotoxicity during the process of cancer cell-vascular endothelium interaction [35]. A complex network of intercellular signaling mechanisms, as well as intracellular signaling cascades, may also regulate these interactions within the metastatic microenvironment [37]. This hypothesis opens a fantastic array of possibilities which, although far beyond the scope of a single paper, deserve further research.

Polyphenols like Pter show potent cytostatic effects [38], [39]. Although, in our experiments we detected lower DNA synthesis capability and cell cycle arrest in S phase (Figure 2A, B), as suggested by others [40], the high reduction in cell number elicited by Pter can be only understood if cell death mechanisms are activated.

Apoptosis has been previously postulated as the main cell death program activated by Pter [38], [40], [41]. However, concentrations unreachable under *in vivo* conditions have been usually tested *in vitro* (≥75 µM). We evaluated the possible contribution of apoptotic cell death by analyzing caspase 3/7 activity and the consequence of their inhibition on the rate of tumor cell growth. Although, caspases are clearly activated by Pter in A375 and A549 cells, pre-incubation with the pancaspase inhibitor zVADfmk was unable to reduce cell death induction (Figure 3A, C). Moreover, HT-29 and MCF7 cells, latter of which do not express caspase-3 [42], failed to activate effector caspases and induce apoptotic caspase-dependent cell death upon Pter treatment (Figure 3A, B). These results suggest that Pter-induced cell death may also occur via caspase-independent mechanism. For this reason we investigated the role of other types of cell death in Pter-induced cytotoxicity.

Macroautophagy (hereafter referred to as autophagy) is an essential conserved cellular process by which lysosomes degrade and recycle damaged organelles and macromolecules and maintain cellular energy levels under nutrient and growth factor deficiency [43], [44]. Although this process regulates the cellular energy homeostasis during nutrient limitation, autophagy may also represent an independent mode of programmed cell death [45], [46]. Indeed, autophagy activation has been proposed as an alternative mechanism of cell death induced by polyphenols [15], [40]. However, contradictory results are presented in the literature [47]. Our data show that Pter induces an accumulation of autophagosomes (Figure 4C) as well as the conversion of cytosolic LC3-I to its lipidated membrane-associated form LC3-II (Figure 4A). Because LC3-II itself is degraded by autophagy, the relative amount of LC3 at a single time point alone, does not necessarily indicate an increase in autophagic flux [22]. P62/sequestosome 1 is a common component of protein aggregates being degraded by autophagy [48], and thus the expression level of this protein serves as a complementary marker for autophagic flux. Pter induced a clear accumulation of P62/sequestosome 1 indicating an inhibition of autophagic flux (Figure 4B).

Microtubules have been suggested to be essential for vesicular and autophagic traffic and consequently, vincristine treatment induces an accumulation of autophagosomes [26], [49]. This accumulation is caused by a failure in the fusion of autophagosomes with lysosomes [50], the increase in autophagosome formation rate [51], or the combination of the two [49]. LC3-II and P62/sequestosome 1 accumulation and the increment in the acidic compartment and lysosomal size suggest that fusion of autophagosomes and lysosomes is alterated. Indeed, natural polyphenols and synthetic analogs are able to inhibit tubulin polymerization acting like anti-mitotic drugs [52]. Lysosomal size alterations by different treatments have been reported to correlate with reduced lysosomal stability and sensitization to non-apoptotic cell death pathways [26], [53]. Accordingly, Pter induced an increase in the size of lysosomes, lysosomal membrane destabilization (Figure 6A, B) and intraluminal content release (Figure 6C, D).

Taken together our data show that Pter-induced cytotoxicity was not primarily the consequence of classic apoptosis, autophagy, or necrosis. Recently, a lysosomal cell death program has been presented as an alternative cell death pathway for demising highly apoptosis-resistant tumor cells [7]. In this sense, we show that Pter promotes the release of lysosomal cathepsins and other hydrolases into the cytosol. Although Res has been shown capable to induce cancer cell death through lysosomal cathepsin D [54] and L [55] release, in our experiments the inhibition of lysosomal cathepsins by pharmaceutical protease inhibitors did not delay the death process (Figure 6E). Therefore, lysosomal membrane permeabilization could be an epiphenomenon of the death process or alternatively, the cytotoxic effect induced by the massive release of lysosomal hydrolases simply cannot be inhibited by blocking the activity of cathepsins D, E, B, L and H. Due to the numerous proteases present inside the lysosomes, and the impossibility to inhibit all of them simultaneously without causing massive alterations in cellular functions, we analyzed the protective effect of HSP70. Interestingly, resistance to Pter treatment correlates with cellular HSP70 levels (Figure 1 and Figure 7A). In fact, inhibition of HSP70 expression (Figure 7B) increased Pter-induced cytotoxicity (Figure 7C) and lysosomal cathepsins release (Figure 7D).

Our results demonstrate that tumor cell death induction elicited by Pter is preferentially mediated through lysosomal membrane permeabilization and depends on HSP70 levels.

## Supporting Information

Figure S1 **Positive controls of apoptosis and zVADfmk inhibition capability.** Activation of apoptosis and the ability of zVADfmk to inhibit the process were determined studying cleaved PARP (Cell Signaling) by western blot. Cells were treated with Camptothecin [A375 (50 nM); A549, HT29, and MCF7 (500 nM)] (A), or Pter (20 µM-50 µM) (B) for 24 and 48 h, in absence or presence of 20 µM pancaspase inhibitor zVAD fmk, which was added 1 h prior to the addition. C) Percentage of apoptotic cells after camptothecin treatment was analyzed by fluorescence microscopy in absence or presence of zVAD fmk.
(TIF)

Figure S2 **Positive controls showing the capability of necrostatin-1 to inhibit necroptotic cell death.** Cells were treated with TNF-α (50 nM) or TNF-α+zVADfmk (20 µM) in presence or absence of 30 µM necrostatin-1 for 48 h to show the capability of necrostatin-1 to inhibit necroptosis. The amount of cell death induced by TNF-α was evaluated by the tripan blue exclusion assay.
(TIF)

## References

[1] Delmas D, Solary E, Latruffe N (2011) Resveratrol, a phytochemical inducer of multiple cell death pathways: apoptosis, autophagy and mitotic catastrophe. Curr Med Chem 18: 1100–1121. 21291372 10.2174/092986711795029708

[2] Asensi M, Medina I, Ortega A, Carretero J, Bano MC, et al (2002) Inhibition of cancer growth by resveratrol is related to its low bioavailability. Free Radic Biol Med 33: 387–398. 12126761 10.1016/s0891-5849(02)00911-5

[3] Pterostilbene. Monograph. Altern Med Rev 15: 159–163. 20807001

[4] Ferrer P, Asensi M, Segarra R, Ortega A, Benlloch M, et al (2005) Association between pterostilbene and quercetin inhibits metastatic activity of B16 melanoma. Neoplasia 7: 37–47. 15736313 10.1593/neo.04337 PMC1490314

[5] Hanahan D, Weinberg RA (2000) The hallmarks of cancer. Cell 100: 57–70. 10647931 10.1016/s0092-8674(00)81683-9

[6] Kroemer G, Jaattela M (2005) Lysosomes and autophagy in cell death control. Nat Rev Cancer 5: 886–897. 16239905 10.1038/nrc1738

[7] Rammer P, Groth-Pedersen L, Kirkegaard T, Daugaard M, Rytter A, et al (2010) BAMLET activates a lysosomal cell death program in cancer cells. Mol Cancer Ther 9: 24–32. 20053771 10.1158/1535-7163.MCT-09-0559

[8] Yang ZJ, Chee CE, Huang S, Sinicrope F (2011) Autophagy modulation for cancer therapy. Cancer Biol Ther 11: 169–176. 21263212 10.4161/cbt.11.2.14663 PMC3230308

[9] Fehrenbacher N, Jaattela M (2005) Lysosomes as targets for cancer therapy. Cancer Res 65: 2993–2995. 15833821 10.1158/0008-5472.CAN-05-0476

[10] Joyce JA, Baruch A, Chehade K, Meyer-Morse N, Giraudo E, et al (2004) Cathepsin cysteine proteases are effectors of invasive growth and angiogenesis during multistage tumorigenesis. Cancer Cell 5: 443–453. 15144952 10.1016/s1535-6108(04)00111-4

[11] Larsen AK, Escargueil AE, Skladanowski A (2000) Resistance mechanisms associated with altered intracellular distribution of anticancer agents. Pharmacol Ther 85: 217–229. 10739876 10.1016/s0163-7258(99)00073-x

[12] Boya P, Kroemer G (2008) Lysosomal membrane permeabilization in cell death. Oncogene 27: 6434–6451. 18955971 10.1038/onc.2008.310

[13] Ostenfeld MS, Hoyer-Hansen M, Bastholm L, Fehrenbacher N, Olsen OD, et al (2008) Anti-cancer agent siramesine is a lysosomotropic detergent that induces cytoprotective autophagosome accumulation. Autophagy 4: 487–499. 18305408 10.4161/auto.5774

[14] Bernhard D, Tinhofer I, Tonko M, Hubl H, Ausserlechner MJ, et al (2000) Resveratrol causes arrest in the S-phase prior to Fas-independent apoptosis in CEM-C7H2 acute leukemia cells. Cell Death Differ 7: 834–842. 11042678 10.1038/sj.cdd.4400719

[15] Opipari AW Jr, Tan L, Boitano AE, Sorenson DR, Aurora A, et al (2004) Resveratrol-induced autophagocytosis in ovarian cancer cells. Cancer Res 64: 696–703. 14744787 10.1158/0008-5472.can-03-2404

[16] Wang Y, Ding L, Wang X, Zhang J, Han W, et al (2011) Pterostilbene simultaneously induces apoptosis, cell cycle arrest and cyto-protective autophagy in breast cancer cells. Am J Transl Res 4: 44–51. PMC3276376 22347521

[17] Ruiz MJ, Fernandez M, Pico Y, Manes J, Asensi M, et al (2009) Dietary administration of high doses of pterostilbene and quercetin to mice is not toxic. J Agric Food Chem 57: 3180–3186. 19292443 10.1021/jf803579e

[18] Ahmad N, Adhami VM, Afaq F, Feyes DK, Mukhtar H (2001) Resveratrol causes WAF-1/p21-mediated G(1)-phase arrest of cell cycle and induction of apoptosis in human epidermoid carcinoma A431 cells. Clin Cancer Res 7: 1466–1473. 11350919

[19] Aggarwal BB, Bhardwaj A, Aggarwal RS, Seeram NP, Shishodia S, et al (2004) Role of resveratrol in prevention and therapy of cancer: preclinical and clinical studies. Anticancer Res 24: 2783–2840. 15517885

[20] Shukla Y, Singh R (2011) Resveratrol and cellular mechanisms of cancer prevention. Ann N Y Acad Sci 1215: 1–8. 21261635 10.1111/j.1749-6632.2010.05870.x

[21] Klionsky DJ, Abeliovich H, Agostinis P, Agrawal DK, Aliev G, et al (2008) Guidelines for the use and interpretation of assays for monitoring autophagy in higher eukaryotes. Autophagy 4: 151–175. 18188003 10.4161/auto.5338 PMC2654259

[22] Mizushima N, Yoshimori T (2007) How to interpret LC3 immunoblotting. Autophagy 3: 542–545. 17611390 10.4161/auto.4600

[23] Denecker G, Vercammen D, Steemans M, Vanden Berghe T, Brouckaert G, et al (2001) Death receptor-induced apoptotic and necrotic cell death: differential role of caspases and mitochondria. Cell Death Differ 8: 829–840. 11526436 10.1038/sj.cdd.4400883

[24] Vanden Berghe T, Vanlangenakker N, Parthoens E, Deckers W, Devos M, et al (2010) Necroptosis, necrosis and secondary necrosis converge on similar cellular disintegration features. Cell Death Differ 17: 922–930. 20010783 10.1038/cdd.2009.184

[25] Kagedal K, Johansson U, Ollinger K (2001) The lysosomal protease cathepsin D mediates apoptosis induced by oxidative stress. FASEB J 15: 1592–1594. 11427496 10.1096/fj.00-0708fje

[26] Groth-Pedersen L, Ostenfeld MS, Hoyer-Hansen M, Nylandsted J, Jaattela M (2007) Vincristine induces dramatic lysosomal changes and sensitizes cancer cells to lysosome-destabilizing siramesine. Cancer Res 67: 2217–2225. 17332352 10.1158/0008-5472.CAN-06-3520

[27] Antunes F, Cadenas E, Brunk UT (2001) Apoptosis induced by exposure to a low steady-state concentration of H2O2 is a consequence of lysosomal rupture. Biochem J 356: 549–555. 11368784 10.1042/0264-6021:3560549 PMC1221868

[28] Kirkegaard T, Roth AG, Petersen NH, Mahalka AK, Olsen OD, et al (2010) Hsp70 stabilizes lysosomes and reverts Niemann-Pick disease-associated lysosomal pathology. Nature 463: 549–553. 20111001 10.1038/nature08710

[29] Asensi M, Ortega A, Mena S, Feddi F, Estrela JM (2011) Natural polyphenols in cancer therapy. Crit Rev Clin Lab Sci 48: 197–216. 22141580 10.3109/10408363.2011.631268

[30] Filippi-Chiela EC, Villodre ES, Zamin LL, Lenz G (2011) Autophagy interplay with apoptosis and cell cycle regulation in the growth inhibiting effect of resveratrol in glioma cells. PLoS One 6: e20849. 21695150 10.1371/journal.pone.0020849 PMC3113895

[31] Cui J, Sun R, Yu Y, Gou S, Zhao G, et al (2010) Antiproliferative effect of resveratrol in pancreatic cancer cells. Phytother Res 24: 1637–1644. 21031621 10.1002/ptr.3157

[32] Kairisalo M, Bonomo A, Hyrskyluoto A, Mudo G, Belluardo N, et al (2011) Resveratrol reduces oxidative stress and cell death and increases mitochondrial antioxidants and XIAP in PC6.3-cells. Neurosci Lett 488: 263–266. 21094207 10.1016/j.neulet.2010.11.042

[33] Davalli P, Rizzi F, Caporali A, Pellacani D, Davoli S, et al. Anticancer activity of green tea polyphenols in prostate gland. Oxid Med Cell Longev 2012: 984219. 22666523 10.1155/2012/984219 PMC3362217

[34] Priego S, Feddi F, Ferrer P, Mena S, Benlloch M, et al (2008) Natural polyphenols facilitate elimination of HT-29 colorectal cancer xenografts by chemoradiotherapy: a Bcl-2- and superoxide dismutase 2-dependent mechanism. Mol Cancer Ther 7: 3330–3342. 18852136 10.1158/1535-7163.MCT-08-0363

[35] Ferrer P, Asensi M, Priego S, Benlloch M, Mena S, et al (2007) Nitric oxide mediates natural polyphenol-induced Bcl-2 down-regulation and activation of cell death in metastatic B16 melanoma. J Biol Chem 282: 2880–2890. 17135264 10.1074/jbc.M605934200

[36] Ortega A, Ferrer P, Carretero J, Obrador E, Asensi M, et al (2003) Down-regulation of glutathione and Bcl-2 synthesis in mouse B16 melanoma cells avoids their survival during interaction with the vascular endothelium. J Biol Chem 278: 39591–39599. 12881529 10.1074/jbc.M303753200

[37] Gassmann P, Haier J (2008) The tumor cell-host organ interface in the early onset of metastatic organ colonisation. Clin Exp Metastasis 25: 171–181. 18058027 10.1007/s10585-007-9130-6

[38] Pan MH, Chang YH, Badmaev V, Nagabhushanam K, Ho CT (2007) Pterostilbene induces apoptosis and cell cycle arrest in human gastric carcinoma cells. J Agric Food Chem 55: 7777–7785. 17696482 10.1021/jf071520h

[39] Mannal PW, Alosi JA, Schneider JG, McDonald DE, McFadden DW (2010) Pterostilbene inhibits pancreatic cancer in vitro. J Gastrointest Surg 14: 873–879. 20140535 10.1007/s11605-010-1164-4

[40] Chen RJ, Ho CT, Wang YJ (2010) Pterostilbene induces autophagy and apoptosis in sensitive and chemoresistant human bladder cancer cells. Mol Nutr Food Res 54: 1819–1832. 20603834 10.1002/mnfr.201000067

[41] Schneider JG, Alosi JA, McDonald DE, McFadden DW (2010) Pterostilbene inhibits lung cancer through induction of apoptosis. J Surg Res 161: 18–22. 20031166 10.1016/j.jss.2009.06.027

[42] Janicke RU (2009) MCF-7 breast carcinoma cells do not express caspase-3. Breast Cancer Res Treat 117: 219–221. 18853248 10.1007/s10549-008-0217-9

[43] Levine B, Klionsky DJ (2004) Development by self-digestion: molecular mechanisms and biological functions of autophagy. Dev Cell 6: 463–477. 15068787 10.1016/s1534-5807(04)00099-1

[44] Mizushima N (2007) Autophagy: process and function. Genes Dev 21: 2861–2873. 18006683 10.1101/gad.1599207

[45] Tsujimoto Y, Shimizu S (2005) Another way to die: autophagic programmed cell death. Cell Death Differ 12 Suppl 2: 1528–1534. 16247500 10.1038/sj.cdd.4401777

[46] Shintani T, Klionsky DJ (2004) Autophagy in health and disease: a double-edged sword. Science 306: 990–995. 15528435 10.1126/science.1099993 PMC1705980

[47] Armour SM, Baur JA, Hsieh SN, Land-Bracha A, Thomas SM, et al (2009) Inhibition of mammalian S6 kinase by resveratrol suppresses autophagy. Aging (Albany NY) 1: 515–528. 20157535 10.18632/aging.100056 PMC2806030

[48] Bjorkoy G, Lamark T, Brech A, Outzen H, Perander M, et al (2005) p62/SQSTM1 forms protein aggregates degraded by autophagy and has a protective effect on huntingtin-induced cell death. J Cell Biol 171: 603–614. 16286508 10.1083/jcb.200507002 PMC2171557

[49] Kochl R, Hu XW, Chan EY, Tooze SA (2006) Microtubules facilitate autophagosome formation and fusion of autophagosomes with endosomes. Traffic 7: 129–145. 16420522 10.1111/j.1600-0854.2005.00368.x

[50] Fengsrud M, Roos N, Berg T, Liou W, Slot JW, et al (1995) Ultrastructural and immunocytochemical characterization of autophagic vacuoles in isolated hepatocytes: effects of vinblastine and asparagine on vacuole distributions. Exp Cell Res 221: 504–519. 7493651 10.1006/excr.1995.1402

[51] Punnonen EL, Reunanen H (1990) Effects of vinblastine, leucine, and histidine, and 3-methyladenine on autophagy in Ehrlich ascites cells. Exp Mol Pathol 52: 87–97. 2307216 10.1016/0014-4800(90)90061-h

[52] Schneider Y, Chabert P, Stutzmann J, Coelho D, Fougerousse A, et al (2003) Resveratrol analog (Z)-3,5,4′-trimethoxystilbene is a potent anti-mitotic drug inhibiting tubulin polymerization. Int J Cancer 107: 189–196. 12949793 10.1002/ijc.11344

[53] Ono K, Kim SO, Han J (2003) Susceptibility of lysosomes to rupture is a determinant for plasma membrane disruption in tumor necrosis factor alpha-induced cell death. Mol Cell Biol 23: 665–676. 12509464 10.1128/MCB.23.2.665-676.2003 PMC151543

[54] Trincheri NF, Nicotra G, Follo C, Castino R, Isidoro C (2007) Resveratrol induces cell death in colorectal cancer cells by a novel pathway involving lysosomal cathepsin D. Carcinogenesis. 28: 922–931. 10.1093/carcin/bgl223 17116725

[55] Hsu KF, Wu CL, Huang SC, Wu CM, Hsiao JR, et al (2009) Cathepsin L mediates resveratrol-induced autophagy and apoptotic cell death in cervical cancer cells. Autophagy 5: 451–460. 19164894 10.4161/auto.5.4.7666

