# Supplementary figures and images for: Pterostilbene-Induced Tumor Cytotoxicity: A Lysosomal Membrane Permeabilization-Dependent Mechanism
Source: PLoS One. 2012 Sep 5;7(9):e44524. doi: 10.1371/journal.pone.0044524 (PMC3434130; doi:10.1371/journal.pone.0044524)

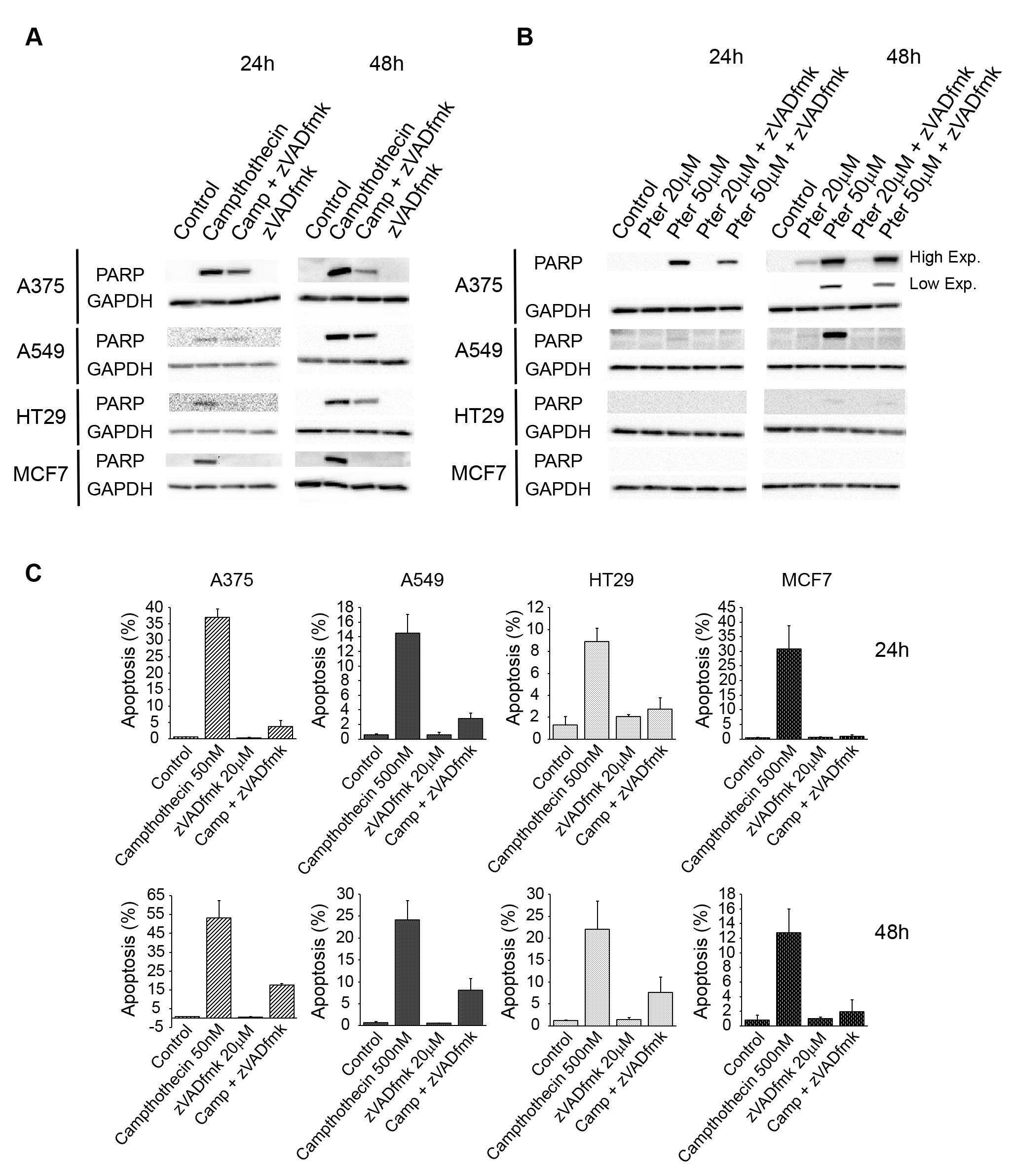

Supplement: Figure S1 — Positive controls of apoptosis and zVADfmk inhibition capability. Activation of apoptosis and the ability of zVADfmk to inhibit the process were determined studying cleaved PARP (Cell Signaling) by western blot. Cells were treated with Camptothecin [A375 (50 nM); A549, HT29, and MCF7 (500 nM)] (A), or Pter (20 µM-50 µM) (B) for 24 and 48 h, in absence or presence of 20 µM pancaspase inhibitor zVAD fmk, which was added 1 h prior to the addition. C) Percentage of apoptotic cells after camptothecin treatment was analyzed by fluorescence microscopy in absence or presence of zVAD fmk. (TIF) [file pone.0044524.s001.tif]

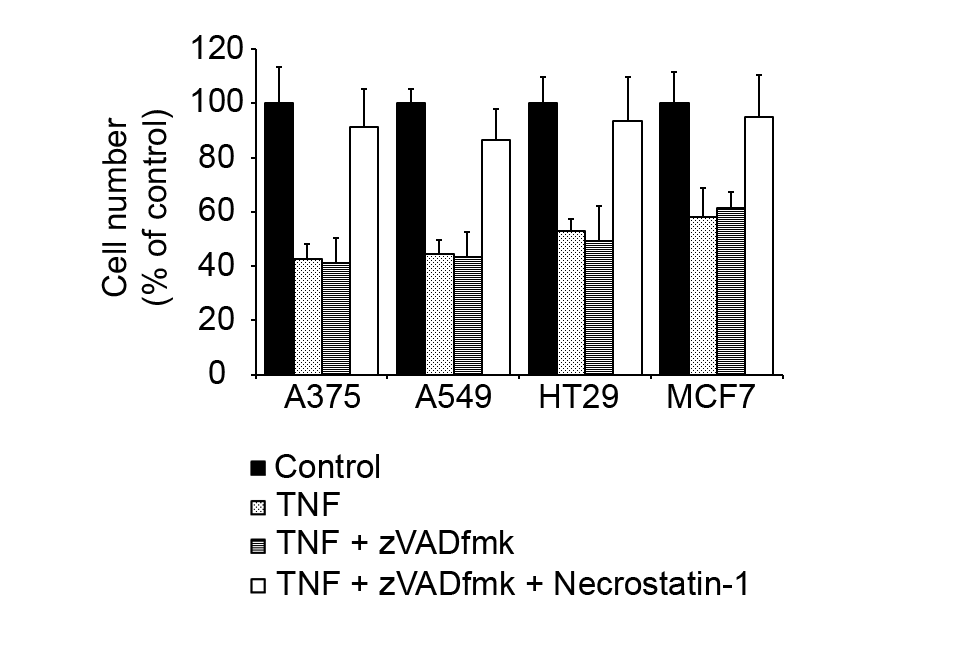

Supplement: Figure S2 — Positive controls showing the capability of necrostatin-1 to inhibit necroptotic cell death. Cells were treated with TNF-α (50 nM) or TNF-α+zVADfmk (20 µM) in presence or absence of 30 µM necrostatin-1 for 48 h to show the capability of necrostatin-1 to inhibit necroptosis. The amount of cell death induced by TNF-α was evaluated by the tripan blue exclusion assay. (TIF) [file pone.0044524.s002.tif]
